# Accreditation as a path to achieving universal quality health coverage

**DOI:** 10.1186/s12992-014-0068-6

**Published:** 2014-10-17

**Authors:** Kedar S Mate, Anne L Rooney, Anuwat Supachutikul, Girdhar Gyani

**Affiliations:** Institute for Healthcare Improvement, 20 University Road, 7th Floor, Cambridge, MA 02138 USA; Healthcare Accreditation Institute, Bangkok, Thailand; National Accreditation Board for Hospitals and Healthcare Providers, New Delhi, India

**Keywords:** Accreditation, Universal health coverage, Quality improvement, Low and middle-income countries, Health financing

## Abstract

As many low- and middle-income countries (LMICs) pursue health care reforms in order to achieve universal health coverage (UHC), development of national accreditation systems has become an increasingly common quality-enhancing strategy endorsed by payers, including Ministries of Health. This article describes the major considerations for health system leaders in developing and implementing a sustainable and successful national accreditation program, using the 20-year evolution of the Thai health care accreditation system as a model. The authors illustrate the interface between accreditation as a continuous quality improvement strategy, health insurance and other health financing schemes, and the overall goal of achieving universal health coverage.

## Introduction

Many low- and middle-income countries (LMICs) are currently pursuing health care reforms to achieve universal health coverage (UHC); however, gaps in health care quality threaten this objective [1,2]. Despite serious resource limitations, there is a growing belief that strengthening health care quality in LMICs can have an important impact on the efficiency, security, and responsiveness of health services, as well as support expansion efforts towards the goal of achieving UHC [3,4]. A 2012 Salzburg Global Seminar convened health leaders from 33 countries to review experiences and identify priority challenges in ensuring healthcare quality and safety in LMICs. The Seminar developed a consensus statement that calls for governments to adopt and promote quality improvement as a cornerstone for better health for all citizens [5]. LMICs have thus rightly sought health systems reforms that not only improve health care coverage but also seek to improve the overall quality of health care services—something we refer to as “universal quality coverage” [6].

The Joint Learning Network (JLN) for Universal Health Coverage is an international consortium of countries implementing health financing reforms aimed at accelerating peer-to-peer knowledge and experience sharing [7]. Nine countries in Africa and Asia are members of the JLN network: Ghana, India, Indonesia, Kenya, Malaysia, Mali, Nigeria, the Philippines, and Vietnam. In 2011, the JLN established a health care quality track which published a summary of methods available to improve health care quality within UHC schemes, including accreditation [8] All nine of the JLN countries either have accreditation schemes or are developing them. At a JLN meeting in Bangkok, Thailand, in April 2013, representatives from all countries shared lessons learned from their accreditation efforts and issued a request for a concise policy document that would offer guidance to country-level decision makers regarding important technical questions that commonly surface.

This document supports future policy development and answers some of the recurrent questions often heard from policy makers, payers and health system leaders: how can accreditation create a culture of continuous quality improvement; what are the key technical choices within accreditation systems and what does the international evidence and experience recommend; what is the role of health care insurance in accreditation; and how can accreditation efforts be leveraged to guarantee basic services in poor and remote communities?

We take these questions in turn, and in each case we summarize the issues and provide the best evidence where possible as it may exist from international sources. There are four sources of information for this evidence: the presentations and proceedings of the April 2013 JLN conference in Bangkok; a review of the literature on accreditation, particularly as it references experience in LMICs; and grey literature and non-peer reviewed source information from leading advocacy and expert organizations like World Health Organization (WHO) and the International Society for Quality in Healthcare (ISQua). Finally, where the data from these other sources were limited, we supplement with the authors’ own experiences from the field. Throughout this document we use ISQua’s definition for accreditation: “A public recognition by a healthcare accreditation body of the achievement of accreditation standards by a health care organization, demonstrated through an independent external peer assessment of that organization’s level of performance in relation to the standards” [9]. We also use the WHO’s definition of UHC: “ensuring that all people can use the promotive, preventive, curative, rehabilitative and palliative health services they need, of sufficient quality to be effective, while also ensuring that the use of these services does not expose the user to financial hardship” [10].

### Key technical areas of accreditation for consideration

#### Legal and governance structure for accreditation

Although important, accreditation is often just one of a number of quality improvement and evaluation strategies, so differentiating it from other regulatory or evaluation mechanisms is essential. Licensure is generally considered a government regulatory responsibility, *designed to set minimum standards* to protect the health and safety of the public. Licensing authorities such as Ministries of Health have the authority to determine which provider organizations can operate, levy fines for deficiencies and, in some cases, even close a substandard provider.

Accreditation, on the other hand, sets standards that are considered *optimal and achievable,* more rigorous than the minimum standards of licensure, and with a stated intent to foster a culture of improvement. In many countries, accreditation is a voluntary recognition program and administered separately from the Ministry of Health, often by a Non-Governmental Organization (NGO) or a quasi-regulatory agency with support and recognition—but at an “arm’s length”—from the government. However, a study by Braithwaite et al. comparing health service accreditation in LMICs with those in higher income countries found that in 60% of respondents from twenty LMICs, accreditation is managed within the Ministry of Health. This is a rare occurrence (only 8% of respondents) in higher income countries. The researchers hypothesized that such a differentiation reflects both a national response to resource limitations in LMICs and an effort to ensure sustainability [11]. Even if administered independently from the Ministry of Health, it is important to note that the accreditation body plays a vital role in advancing the Ministry’s overall quality and safety agenda.

Legislation to establish the legal structure and scope of the accrediting body is an important first step which can ensure the sustainability of the accreditation program. The survey profile of 44 global accreditation organizations (AOs) published by Shaw et al. in 2013 demonstrated that two-thirds of responding AOs were formally authorized by national legislation, official decree, or both. Government strategies tended to be associated with legislation. Those that were not formally authorized by law tended to be the larger, more mature AOs, such as in Australia, USA, England, and South Africa, which were originally organized as independent Non-Government Organizations (NGOs) [12]. Regardless of whether it is mandated by legislation, there should be a legal charter, articles of incorporation, or bylaws that clearly describe the role of the accrediting body, its governance, scope of responsibility and authority, and relationship to government oversight of quality. For example, will the government licensing agency accept an accreditation award in lieu of doing its own evaluation? Will the accreditation program be mandatory or voluntary? Many countries have opted for a voluntary approach to accreditation, thus differentiating it from licensure (mandatory) and intending it to recognize a higher level of achievement. Regardless, there should be close collaboration between the licensing agency and the accreditation body, in order to reinforce compliance with regulatory requirements and better enable sustainability of the AO [13-17].

Governance of the accrediting body frequently represents a credible cross-section of health care professionals (such as professional societies, medical or nursing boards), the public at large, and other stakeholders such as financing agencies, industry, NGOs, and/or academic institutions. The international survey of AOs by Shaw et al. reflected a preponderance of healthcare clinicians, especially physicians, in governance roles. Government regulators were represented in approximately half of the responding AOs, while insurers and patients and families were represented in about a third of the 44 responding AOs [12]. In order for the eventual accreditation award to be credible, the governing body’s objectivity must be above reproach. Therefore, any potential conflicts of interest must be addressed up front through rigorous governance screening and selection, policies, and codes of ethical conduct.

#### Standards development and management

The heart of any accreditation program lies in the reliability, validity, measurability, and objectivity of its standards. Accreditation standards must encourage improved performance, while at the same time being achievable and not overly prescriptive. An excellent framework for guiding the standards development process is available free of charge from ISQua through its recently revised *International Principles for Healthcare Standards* [18].

Standards development typically begins with a thorough literature review to assess the current set standards from within the country (e.g., licensing, food and drug safety regulations, building and fire codes), as well as other national programs or global accreditation organizations. A 2009 study of 18 European accreditation organizations demonstrated a movement from a traditional collegial model of accreditation toward more of a semi-regulatory model that incorporates minimal licensing and public safety standards with aspirational standards for continuous improvement [19]. The standards should address all critical elements of quality and safety in the specific health care provider category, including patient care processes such as assessment and treatment, medication management and safety, infection prevention, blood usage, diagnostic services such as laboratory and radiology, anesthesia and surgical services, patient education, and continuity of care. In addition, critical management components must be addressed, including governance and leadership, financial management, staff credentialing and human resource management, building safety, patient rights and ethics, medical records and information management, and quality and safety management. Wherever possible, the accreditation program should engage subject matter experts in the development and review of new or revised standards. One approach is to use advisory structures that function in a “virtual” environment, through electronic document review and videoconferencing [18,20].

Before the draft standards are finalized, it is critical to seek stakeholder feedback from as many sources as possible, including providers, health care professional societies, the public, employers, and consumer advocacy groups. This process of stakeholder engagement helps to ensure that the final version of the standards will be seen as credible, understandable, and rooted in good clinical and management practices. We recommend a pilot test of any new standards at a sampling of provider organizations. The pilot should use an objective on-site survey or evaluation process, to confirm that the standards can be measured in a comprehensive, reliable, and consistent manner. Adjustments can then be made to the standards or survey process before the final standards are launched. Periodic revision is also important. The ISQua standards require a thorough standards review and revision process at least every four years.

#### Accreditation program management

Leadership of an effective accreditation program includes operational management; surveyor selection, training, credentialing, and ongoing supervision and support; operating policies and procedures; design and implementation of a credible and objective evaluation process that includes sampling criteria; the application, staffing, and scheduling processes for conducting the on-site survey; field education to providers in the standards, evaluation process, and quality improvement strategies; financial management; and information management [21]. The challenges to successful program management in resource-limited settings are considerable. For example the challenges may range from corruption to poor information management (thus making data collection and evaluation especially difficult) to knowledge gaps about quality and safety among health care workers to difficulties in surveyor travel to remote settings of healthcare delivery. Research by Sax and Marx of accreditation development in one province in Pakistan demonstrated that a major challenge was the establishment of a well-managed, transparent accreditation agency able to lead processes such as training and support for peer surveyors. This study also identified that an understanding of local change mechanisms and cultural practices is important in designing a sustainable accreditation approach [22].

Well-trained, knowledgeable, and objective surveyors are critical to the design of any successful accreditation program. Among the 20 LMIC countries represented in the Braithwaite et al. survey of global accreditation organizations, almost 90% indicated they have instituted a process by which surveyors are formally certified after successful completion of training [11]. In addition to their knowledge of the standards and evaluation processes, surveyors must possess a high degree of personal and professional integrity as well as an ability to teach, inspire, and motivate provider organizations to a high level of performance. If surveyors are viewed as biased or punitive, the goal of the accreditation program in stimulating a culture of improvement will fail. Some accreditation programs have added a public or consumer representative to the evaluation team, with a particular focus on how the provider organization addresses patient needs such as wait times, food quality, privacy, and patient rights.

Valid data are also essential, thus warranting the design of an efficient and effective data collection and management function. This function must include the initial application for accreditation, the profile of the institution to be accredited, the on-site survey findings, other indicator data, as well as the accreditation decision itself.

When the accreditation program captures data about provider performance during the on-site evaluation, this data can be used not only to make assessments about the individual provider over time, but can be aggregated to give a national view as well. This aggregate performance data can inform larger health policy decisions, funding, and education. For example if the aggregate hospital accreditation data reveals a systemic problem with antibiotic overuse and misuse at a national level, policy changes might modify he national formulary and develop consistent clinical policies for appropriate antibiotic stewardship throughout the country.

#### Accreditation decision process

Valid, reliable, and transparent decision criteria should be employed in making the determination of an accreditation award, so that the “rules” are known and understood by all and the credibility of the accreditation program is supported. The criteria and the threshold for achieving accreditation should aim to both recognize outstanding performance and differentiate weak performance, while still motivating provider organizations toward a culture of continuous improvement. Some accreditation programs do that through implementing a tiered or “step-wise” approach to accreditation along an improvement continuum. The accreditation award is typically renewed via a process of an on-site survey every 2–4 years.

The criteria must also address what happens when an accredited provider experiences a significant quality or safety incident, changes ownership, or embarks on a major construction project. If the accreditation program withdraws accreditation for some reason, under what circumstances can the provider reapply? The accreditation program’s policies should specify if, and how, the results of the accreditation award will be made public and/or reported to the Ministry of Health. This is especially critical in those cases where the surveyors identify a serious threat to public health or safety. The accreditation program typically does not have the legal authority to close a facility, whereas the Ministry of Health could do so in those rare circumstances.

Accreditation, because it involves an “external peer review” that is intended to place a value judgment on the quality, safety, and cleanliness, of an institution, has often been regarded as a form of summative evaluation rather than a mechanism for formative learning. However, as described in the developmental milestones of Thailand’s Healthcare Accreditation Institute (Table 1), accreditation ideally should mature towards a more formative, continuous learning environment.

**Table 1:** **Milestones of the developmental journey of health care accreditation in Thailand** [23]

**Year**	**Initiatives**
1993-1995	Pilot project of TQM in public hospitals to learn how quality improvement tools and concept can be applied to health care.
1995-1996	Development of first hospital accreditation (HA) standards, continuous quality improvement concept being incorporated.
1997-1999	Standards implementation as a research and development project, emphasized a multidisciplinary team approach. Lab and pharmacy standards are used.
1999	*First National Forum on Quality Improvement and Accreditation* is held, and continues annually as a forum for experience sharing and updating knowledge.
1999	Institutionalization of the project, The Institute of Hospital Quality Improvement and Accreditation (HA) was founded under the umbrella of the Health Systems Research Institute.
2001	The Universal Health Coverage (UHC) policy launched in Thailand, setting the expectation for a quality health care system.
2003	The HA program started a stepwise recognition program to gain acceptance and expand coverage, encouraging continuous improvement considering potential and limitation of each hospital.
2003	Health Promoting Hospital (HPH) standards and accreditation program were developed.
2006	First HA Patient Safety Goals were developed and instituted.
2006	Integration of HA & HPH standards, using National Quality Award framework.
2009	Introduction of standards addressing spirituality into quality improvement.
	The HA/HPH Standards were accredited by ISQua.
	The accreditation body was transformed to be The Healthcare Accreditation Institute (Public Organization), an autonomous government agency.
2010	Quality Learning Networks, empower accredited hospitals to give assistance to their peer hospitals.
2012	Community of Practice for high-risk services.

#### Financing for accreditation

Sustainability of accreditation is largely dependent on sufficient financing, not only for the initial development costs, but for ongoing governance and operations, surveyor training and management, education to provider organizations, and potentially, to make needed improvements within the provider organizations themselves. Therefore, it is useful to project a 5-year financing plan; for example, the initial (years 1 and 2) development costs may be supported by the Ministry of Health or by a donor or funding agency, but after launch, ongoing financial viability will need to be carefully considered. Will the provider organizations be assessed a fee for the accreditation visit and related travel expenses? Especially in the start-up phase when there are a smaller number of participating providers, how will these fees support ongoing operations until the program becomes self-sustaining? Fees generated from related education programs are often a good revenue source to support the operations of the accreditation body. Still, Shaw et al’s survey of 18 European accreditation organizations in 2009 identified that the size of the country’s population and thus number of potential accreditation customers can have a significant impact on the long-term viability of the accreditation operation. After initial start-up funding, the ongoing operations will need to be supported by institutional customers, and in a country with a small number of healthcare provider organizations, these costs can be considerable [19].

In addition to the financial support to the accrediting body, it is also critical to consider how provider organizations will be supported in their accreditation and improvement efforts. For example, how will needed improvements which involve capital costs such as facility renovation or the purchase of large equipment be addressed? Without this consideration, the accreditation program runs the risk of irrelevance, as it promotes standards and expectations that are not achievable. Some countries have found it helpful to secure external funding support for at least 4–5 years as the accreditation program is developed and launched. Financial support can even extend to education and technical assistance to pilot groups of provider organizations that serve as demonstration projects and models for meeting the standards.

#### Incentives for accreditation

In order for health care leaders and professionals to embrace the philosophy of accreditation, they must view it as making a discernible difference in quality and safety as well as a sound business decision. For the former, data on valid indicators such as infection rates and maternal mortality – from baseline through the entire improvement and accreditation journey – can engage clinical leaders and staff who are motivated by a genuine interest in attaining improved outcomes. An international survey of 44 global accreditation organizations found that more than 80% indicated “quality improvement” as the primary motivator for accreditation, although commercial incentives and benefits also played an important motivating role [12].

Linking accreditation to securing favorable bank loans and payment terms as well as other forms of recognition such as reimbursement differentials, participation in insurance schemes, “preferred provider” status from employers, and designation as medical travel destinations have been effective mechanisms for making the “business case” for accreditation [24-26]. The 2009 survey of European accreditation organizations, discovered that “the uptake and stability of voluntary accreditation are largely dependent on tangible commercial advantage to accredited institutions, either by increasing market share or by direct funding” [19]. In India, the CGHS (Central Government Health Scheme) has made provision to provide 15% more remuneration to hospitals accredited by the National Accreditation Board for Hospitals and Healthcare Providers (NABH) [27].

#### Interface of insurance and accreditation

Increasingly, insurers and employers are relying on an objective and credible accreditation award as a prerequisite for provider participation in their health care reimbursement programs. In some instances this is called “empanelment”, to represent a standard/criteria driven process whereby providers are selected to participate in the insurance program. This is frequently done in lieu of the insurer or employer conducting its own on-site evaluation or performance data collection and analysis. Examples include India, Brazil, and Costa Rica [28]. In these situations, while accreditation remains a voluntary process, the financial incentives can be powerful enough that it becomes “quasi-mandatory” and seen as essential to good business for a successful and reputable health care provider. Some employers and insurers – even some government payers -- provide extra motivation by rewarding good performance with a higher reimbursement rate as compared to unaccredited providers [29].

Ideally, the insurer or payer would recognize the considerable achievement of performance that accreditation represents and would not duplicate or contradict prevailing national accreditation standards or data collection requirements through its own quality evaluation criteria. That creates a useful symbiosis whereby the insurer or payer can justifiably rely on an objective third party (the accreditation body) for a credible evaluation, and the provider in turn is motivated to continuously improve its performance by virtue of receiving the financial benefit of receiving new patients with insurance coverage. This virtuous cycle points to the need for the accrediting body to engage insurers and payers at the front end of the development process, as well as to secure their buy-in and support for the sustainability of the accreditation program. Many mature accreditation organizations now offer some degree of “regulation by proxy”, as a third party assessor of compliance with regulation on behalf of a government or payer, thus reducing the burden of inspection by multiple bodies [12].

Insurers can also play a critical role in data and knowledge sharing with the accreditation program, in the forms of guidelines, protocols, tools, and aggregate data on provider performance. Many insurers and corporations will help to fund educational programs and resources about the accreditation standards, quality, and safety, and thus can become strong allies in supporting accreditation in a country.

#### Accreditation as a progressive improvement tool

A common question at the start of any accreditation development initiative is often, “Can one size really fit all?” In other words, is it possible to develop a single set of standards which apply to a common provider type (e.g., hospital, clinic, clinical laboratory) within a country, ranging from a large tertiary hospital to a small and rural district facility? Some countries and programs have used a progressive and step-wise approach to accreditation for *all* providers, starting first with more basic structural standards for hand-washing and facility safety and then “raising the bar” with more complex and outcome-focused standards. If this approach is adopted, it is useful to communicate an overall plan for the kind of standards progression and expected timeframes for achievement (e.g., more progressive standards will be introduced every 2–3 years). That way all providers will understand this up front as both encouraging and expecting improved performance over time. The public recognition given to achieving even the most basic level of performance at the start of the national accreditation system can be a valuable building block in creating a national culture of quality improvement. The case study in Table 2 highlights some of the lessons learned from the national improvement journey in Thailand.

**Table 2:** **Key principles from the experience in Thailand in how to create a culture of continuous quality** [23]

I.	Describe the ingredients needed in the early days to start on the right path
II.	Work closely with professional associations (with roles of setting guidance, giving advice, and information sharing)
III.	Demonstrate benefits of accreditation and quality improvement for staff
IV.	Make it voluntary. Offer opportunities for doctors and hospitals to participate; don’t tell them that it is mandatory.
V.	Focus on coaching/learning, not inspection/audit. Don’t focus too much on pass/fail, but instead, what needs to be done to improve.
VI.	Focus also on knowledge activities (e.g., large Thai annual quality educational meeting).
VII.	Use recognition of hospital staff as well as offer opportunities to optimize their potential.
VIII.	Make it fun, inspiring!
IX.	Have payer organizations provide incentives (e.g., health insurance and social security pays more to accredited hospitals).
X.	Treat the organization/hospital as living system – self-organizing, learning.

Another approach to address basic levels of quality improvement, especially in smaller and resource-poor facilities, is to develop an accreditation system that takes into account that not all providers will ever be able to meet the same set of standards. In the U.S., this approach is seen with Critical Access Hospitals, providers in rural areas with a licensed capacity of 25 beds or less; these hospitals are required to meet a different, less complex set of standards than their tertiary care counterparts [30]. Some accreditation programs around the world use a common set of basic standards for all, and then add standards components for complexity and specialization, such as for pediatric or psychiatric hospitals.

A third approach to progressive accreditation can be in the design of the accreditation program, both in its standards as well as its criteria and threshold for achieving accreditation. For example, a single standard for implementing an infection control program may receive an entry-level score when the provider implements a required structure such as a policy or procedure, and then progress to a full accreditation status when its infection control program is fully implemented and demonstrates through its data that it is effective in controlling infections. This approach adopts a Structure-Process-Outcome framework to improvement by recognizing the beginning steps of implementing structures such as policies or committees, yet motivates and stretches providers to achieve an even higher level of performance.

## Conclusion: realizing the promise of accreditation

**Figure 1:**
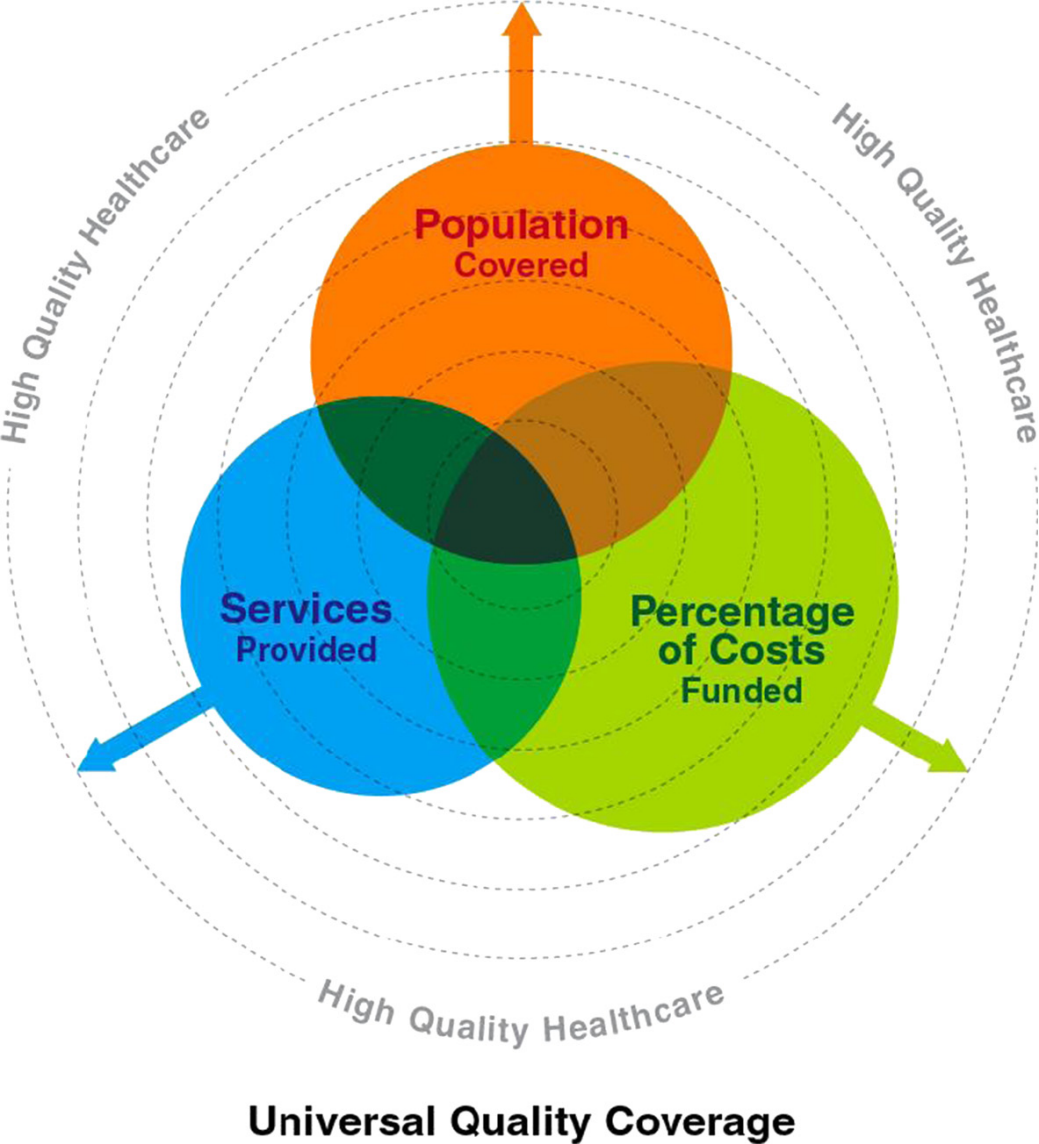
**Four dimension of universal quality coverage.**

The true goal of UHC schemes is to provide universal access to high-quality health care services. This “universal quality coverage” will only be realized if quality-enhancing mechanisms like accreditation can be successfully implemented in LMIC settings. To date, efforts to depict how UHC scheme will work have focused on three dimensions: expanding coverage, describing the benefits covered and financial risk protection that the coverage scheme offers. Descriptions of the quality of care that the coverage scheme offers has not been well addressed. We propose a new image moving from the model proposed in the WHO Report on UHC to the model shown in Figure 1 where coverage, benefits and financial risk protection are all represented as overlapping and expanding balloons. They reach outwards through gradations of quality of health services represented by the dashed lines towards the outer limit of high quality health care services.

Accreditation is among the most important strategies LMICs utilize to improve quality of care. The guidance provided in this paper is intended to assist governments, health system planners, and decision makers to make useful investments in accreditation or other quality-enhancing strategies as they pursue UHC. No two countries will be alike and thus, the important choices described above will vary from one context to the next.

No matter what type of accreditation design is pursued, the results from the external peer-review process should be fed into a continuous improvement effort that allows institutions to remediate gaps in structures, processes and outcomes and to ensure that where patterns in health system defects are recognized (as in the antibiotic overuse example described above), national-level action can be taken to improve care.

**Figure 2:**
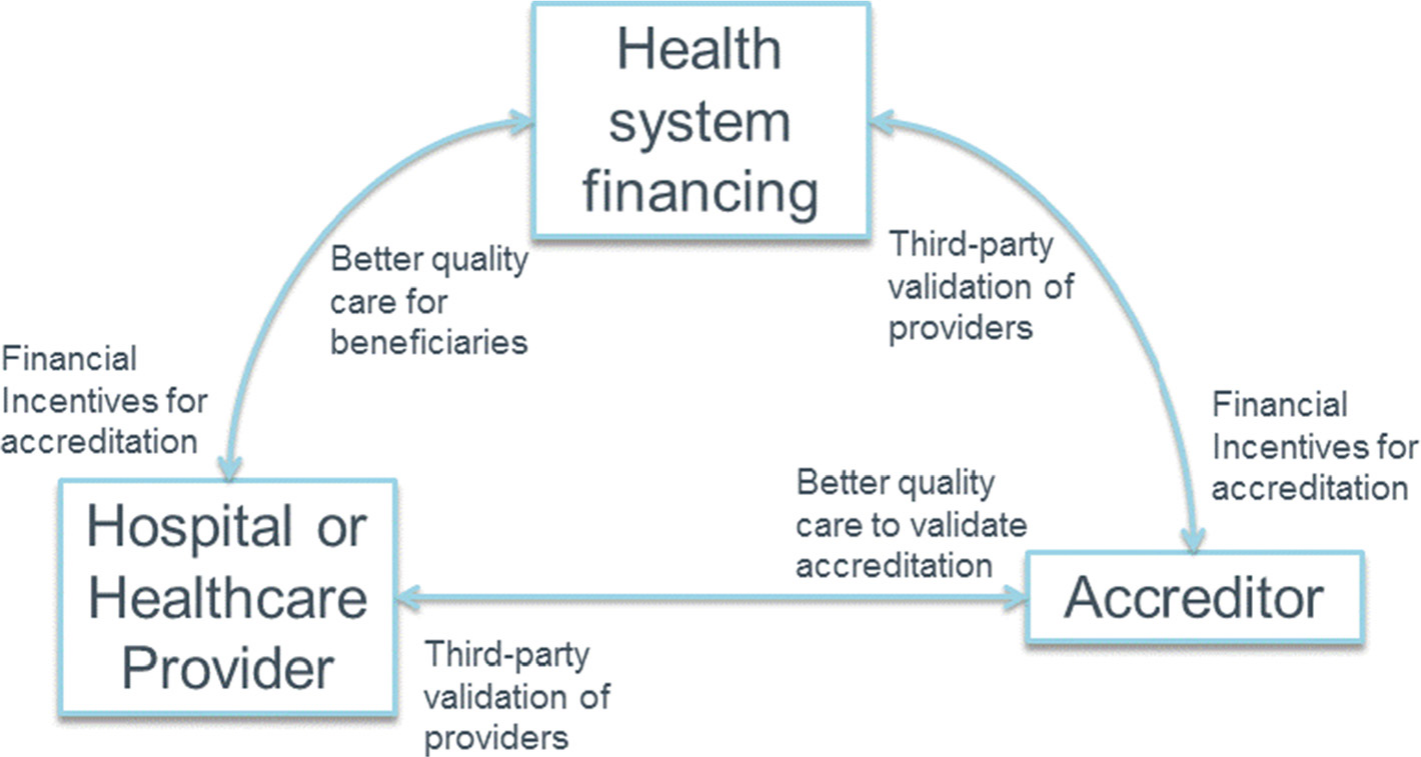
**Reinforcing relationships between accreditation, financing and providers.**

Accreditation and health system financing for universal coverage have the potential to be particularly reinforcing if deployed correctly. At the most basic level, payers for UHC could reinforce accreditation standards by providing the needed financial incentives to institutions to seek accreditation. In turn, accreditation can provide payers with the independent third-party evaluation of health care quality that they need to make sound decisions about which institutions and professionals to include in their reimbursement schemes. (Figure 2) On the other hand, lack of coordination between them will lead to confusion in the marketplace about which standards and protocols to follow, which measures or indicators to report, and which incentives to follow.

Achieving “universal quality coverage” will require alignment between government, payers and accreditors. There are many choices that opinion leaders, policy makers, and regulators face as they design health care systems to ensure high-quality service. This paper reviews some of the better practices that are available; however, much remains unknown about the role and ideal design of accreditation systems that will accelerate achievement of universal quality coverage. By identifying the gaps in our understanding, we hope to not only inform decision makers but also researchers interested in pursuing further study to help bring clarity to the field.
